# Anthocyanin rich extract of *Brassica oleracea* L. alleviates experimentally induced myocardial infarction

**DOI:** 10.1371/journal.pone.0182137

**Published:** 2017-08-01

**Authors:** Sarmita Jana, Dipak Patel, Shweta Patel, Kapil Upadhyay, Jaymesh Thadani, Rahul Mandal, Santasabuj Das, Ranjitsinh Devkar

**Affiliations:** 1 Phytotherapeutics and Metabolic Endocrinology Division, Department of Zoology, Faculty of Science, The M.S. University of Baroda, Vadodara, India; 2 Ecotoxicology lab, Jai Research Foundation, Vapi, India; 3 Biomedical Informatics centre, National Institute of Cholera and Enteric Diseases, Kolkata, India; University of Hyderabad, INDIA

## Abstract

Cardioprotective potential of anthocyanin rich red cabbage extract (ARCE) was assessed in H_2_O_2_ treated rat neonatal cardiomyoblasts (H9c2 cells) and isoproterenol (ISO) induced rodent model of myocardial infarction. H_2_O_2_ treated H9c2 cells recorded cytotoxicity (48–50%) and apoptosis (57.3%), the same were reduced in presence of ARCE (7–10% & 12.3% respectively). Rats pretreated with ARCE for 30 days followed by ISO treatment recorded favourable heart: body weight ratio as compared to ISO treated group. Also, the mRNA levels of enzymatic antioxidants (*sod* and *catalase*) and apoptotic genes (*bax* and *bcl-2*) in ARCE+ISO treated group were similar to the control group suggesting that ARCE pretreatment prevents ISO induced depletion of enzymatic antioxidants and apoptosis. Histoarchitecture of ventricular tissue of ISO treated group was marked by infracted areas (10%) and derangement of myocardium whereas, ARCE+ISO treated group (4.5%) recorded results comparable to control (0%). ARCE+ISO treated group accounted for upregulation of *caveolin-3* and *SERCA2a* expression as compared to the ISO treated group implying towards ARCE mediated reduction in membrane damage and calcium imbalance. Molecular docking scores and LigPlot analysis of cyanidin-3-glucoside (-8.7 Kcal/mol) and delphinidin-3-glucoside (-8.5 Kcal/mol) showed stable hydrophobic and electrostatic interactions with β_1_ adrenergic receptor. Overall this study elucidates the mechanism of ARCE mediated prevention of experimentally induced myocardial damage.

## Introduction

Cardiovascular diseases (CVDs) cause significant morbidity and continue to remain the leading cause of death globally. Myocardial infarction (MI) is the most prevalent type of CVD wherein loss of cardiomyocytes due to apoptosis is at the epicentre of its pathogenesis [1]. Reactive oxygen species (ROS) mediated depletion of intracellular antioxidants, lipid peroxidation, modification of structural proteins and DNA damage precede apoptosis. Therefore, reduction of intracellular ROS is one of the key targets of research to regulate apoptotic cascade in cardiomyocytes [2]. Rat cardiomyocytes when treated with H_2_O_2_, undergo cellular damage due to production of free radicals wherein, the sequences of events are similar to an oxidatively stressed myocardium. Isoproterenol (ISO) is a synthetic β adrenergic agonist that causes myocardial hyperactivity, coronary hypotension hypoxia [3], calcium overload and infract like necrosis [4]. Therefore, H_2_O_2_ induced oxidative stress and ISO induced myocardial infarction models are used to assess cardioprotective potential of the test compounds [5, 6]

Anthocyanins (a member of flavonoid family) are polyhydroxyl and polymethyl derivatives of flavynium salts that have been extensively reported to manifest therapeutic properties against alzheimer’s disease [7], hyperlipidaemia [8], hyperglycaemia [9], cardiovascular diseases [10], diabetic retinopathy [11] and in lowering blood pressure [12]. Red cabbage (*Brassica oleracea* L; Family-Brassicaceae) is a commonly consumed functional food in Asia and Europe due to its low calorie-high fibre composition [13]. It is also a rich source of anthocyanins such as cyanidin-3-diglucoside-5-glucoside and their various acylated derivatives [14, 15]. Reports on its hepatoprotective [16], membrane stabilizing [17] and neuroprotective [18] potentials have been published wherein, therapeutic potential of red cabbage has been attributed to high content of anthocyanins. Previous studies in our lab had reported that co-supplementation of Anthocyanin rich Red Cabbage Extract (ARCE) prevents cardiac and hepatic oxidative stress in atherogenic diet fed rats [19] and improves mitochondrial membrane potential in oxidatively stressed cardiomyoblasts [20]. Though, consumption of anthocyanin in reducing cardiovascular risks and myocardial infarction [21] has been reported, ARCE has not been investigated in detail for its cardioprotection.

Extensive reports on cardioprotective potential of anthocyanins and leads from our previous study on ARCE prompted us to initiate a detailed investigation. Study showcased herein, assesses the mechanism of cardioprotective potential by ARCE via *in vitro*, *in vivo* and *in silico* models.

## Materials and methods

### Materials

All chemicals of molecular biology grade were purchased commercially. Methanol, dimethyl sulfoxide (DMSO), 3-(4,5-dimethylthiazol-2-yl)-2,5-diphenyl tetrazolium bromide (MTT) were purchased from Sisco Research Laboratory Pvt. Ltd. (Mumbai, India). Triphenyl tetrazolium chloride (TTC) stain, hematoxylin, eosin and isoproterenol (ISO) were purchased from Sigma-Aldrich (St. Louis, MO, USA). Fetal bovine serum (FBS), Dulbecco's Modified Eagle's Medium (DMEM), trypsin phosphate versene glucose (TPVG) and antibiotic-antimycotic solution were purchased from Hi-media Laboratories (Mumbai, India). Annexin V-Alexa 488, Propidium Iodide (PI), TRIzol reagent, DreamTaq Green master mix and SYBR select master mix were procured from Invitrogen (CA, USA). iScript cDNA synthesis kit was purchased from Bio-Rad (CA, USA). ENZOPAK Creatine Kinase-Myocardial b fraction (CK-MB) kit was purchased from Reckon Diagnostics (Vadodara, Gujarat). RNAlater stabilizing solution was purchased from Ambion Inc. (USA).

### Preparation of ARCE

Red cabbage (*Brassica oleracea* L. var. Capitata f. rubra DC.) was procured from Spencer’s mall, Vadodara, Gujarat, India (22° 19’ 21” N, 73° 10’ 32” E), identified and authenticated by Dr. Vinay Raole, Department of Botany and voucher specimen (accession no. 213) was submitted to departmental herbarium (BARO), The M. S. University of Baroda, Vadodara, Gujarat. Fresh red cabbage was chopped into small pieces and extracted using methanol: water: HCl (50:50:1) solvent system. The resultant extract was dried in rotatory evaporator at 40°C, cooled at room temperature and stored at 4°C till further analysis [22]. The resultant yield (7.1% w/w) was diluted with distilled water and the total Anthocyanin content was measured spectrophotometrically using molar extinction coefficient of cyanidin-3,5-diglucoside (26,300 M^-1^ cm^-1^).

### Identification of anthocyanins in ARCE

Anthocyanins were identified by Thin Layer Chromatography (TLC) and gas Chromatography/ Mass spectroscopy (GC/MS). Briefly, methanolic solution of ARCE (40mg/ml) was subjected to TLC (Silica gel 60 F_254_) and developed in a pre-saturated chamber of Ethyl acetate: Glacial acetic acid: Formic acid: H_2_O (10: 1.1: 1.1: 2.6). Plates were dried at room temperature and bands were scraped using a clean scalpel. The contents were dissolved in 5 ml methanol and filtered (Whatman Filter paper No. 1). Filtrates were dried in rotatory evaporator (40°C), cooled to room temperature and stored at 4°C till further analysis. Sample (2.5 mg/ml methanol) was injected through pre-filter unit using Helium gas (99.9% gas carrier) with a flow rate of 1ml/min. The column (30m PE-5ms) temperature was held at 60°C for 5min and then increased up to 280°C at the rate of 10°C/min. Mass spectra were scanned from 10 to 610 u.

### Experimental design

#### Maintenance of H9c2 cells

Rat cardiomyoblasts (H9c2 cells) were procured from National Centre for Cell Science (NCCS, Pune, India) and maintained at 37°C with 5% CO_2_ in DMEM (10% FBS and 1% antibiotic antimycotic solution). Cells were trypsinized using 1X TPVG at every third day. The study was grouped as Control (untreated cells), ARCE (treated with 250 μg/ml ARCE for 24 h), H_2_O_2_ (100 μM for 12 h) and ARCE+ H_2_O_2_ (pre-treated with ARCE for 24 h followed by H_2_O_2_ for 12 h).

#### Cytotoxicity assay

H9c2 cells were seeded in 96 well plate (10^4^ cells/well), allowed to grow overnight and were treated as mentioned above. MTT (5 mg/ml) was added in each well and incubated in dark for 4 h. The resultant purple formazan crystals were dissolved in DMSO (150 μl/well) and absorbance was measured at 540nm using ELX800 universal Microplate Readers (Bio-Tek instruments, Inc., Winooski, VT) and % cytotoxicity was calculated with respect to control.

#### Apoptosis assay

Cells (1×10^4^) of various groups were trypsinized, centrifuged and washed with PBS. Control and treated cells were stained with AnnexinV-Alexa 488 and Propidium Iodide for 15 min at 37°C in dark [23] and subjected to flow cytometric analysis. The data were acquired using BD FACSAria^™^ III (BD Biosciences, USA).

#### Experimental animals

Adult albino male Charles foster rats (n = 36, 160–180 g) were obtained from Department of Biochemistry, The M.S. University of Baroda, Vadodara, India. Throughout the study, rats were maintained in clean polypropylene cages and controlled conditions (23±2°C, LD 12:12 and 45–50% humidity with food and water *ad libitum*) as per standard guidelines of Committee for the Purpose of Control and Supervision of Experiments on Animals (CPCSEA). The experimental protocol (P.N.3approval no. 827/ac/04/CPCSEA) was approved by the Institutional Animal Ethics Committee (IAEC) and the Committee for the Purpose of Control and Supervision of Experiments on Animals (reg. no. 827/ac/04/CPCSEA) of the Department of Zoology, The M. S. University of Baroda, Vadodara, Gujarat, India. Rats were acclimatized for 10 days prior to setting up of the experiment. During the entire period of study, the health condition of rats was closely monitored for any possible injury and abnormal behaviour. Weekly records of food consumption and body weight were maintained.

#### Isoproterenol (ISO) model of myocardial infarction

Rats were randomly divided into three groups of six animals each and dosed via gastric intubation as follows. Group 1 (Control): normal saline daily for 30 days. Group 2 (Disease control): normal saline daily for 28 days followed by Isoproterenol (ISO: 85 mg/kg body weight *s*.*c*.) on 29^th^ and 30^th^ day. Group 3 (ARCE+ISO): ARCE (250 mg/kg body weight) daily for 28 days and Isoproterenol (ISO: 85 mg/kg body weight *s*.*c*.) on 29^th^ and 30^th^ day. At the end of the experimental period (31^st^ day) rats were fasted overnight (12h). The next day, rats were anaesthetised with 1% pentobarbital sodium (40 mg/kg, *i*.*p*.) and blood samples were collected from retro-orbital sinus puncture. Blood samples were centrifuged at 3000 rpm for 10 min at 4°C and plasma was stored at -20°C till further analysis. Animals were sacrificed by decapitation and whole hearts (6 per group) were excised and weighed. Ratio of heart: body weight (cardiosomatic index) was calculated [24]. Some part of the ventricular tissue (~50 mg) was cut from each heart and fixed in RNAlater stabilization solution at -20°C. The remaining ventricular tissue was horizontally divided into two parts for histopathology and study of infracted area respectively.

#### Gene expression studies

Cardiac tissue samples collected in RNAlater stabilization solution were washed with DEPC water. Total RNA was isolated using TRIzol reagent. cDNA was synthesized by reverse transcription of total RNA (1 μg) using iScript cDNA Synthesis kit. Further, mRNA levels of enzymatic antioxidants (superoxide dismutase; *sod* and *catalase*), apoptotic genes (*bax* and *bcl-2*), myocardium specific caveolae protein (*caveolin-3*), and sarco/endopalsmic reticulum calcium ATPases (*SERCA2a*) with *GAPDH* as an internal control were evaluated by quantitative PCR as elucidated herein.

Quantitative PCR analysis (QuantStudio 12K Flex, Life Technologies, CA, USA) was performed using SYBR Select Master Mix. The reaction mixture consisted of cDNA (0.8 μl), forward and reverse primers (0.4 μl each), SYBR green master mix (5 μl) and ultrapure water (3.4 μl). Melting curve of each sample was measured to ensure the specificity of the products. The data were normalized to the internal control *GAPDH* and analysed using 2^-ΔΔCT^method [25]. Primers used for this study are listed in Table 1.

**Table 1:** Primers for quantitative PCR.

Gene Name	Accession number	Forward Primer (5’→3’)	Reverse Primer (5’→3’)	Product length
*GAPDH*	NM_017008.4	actttggcatcgtggaaggg	acttggcaggtttctccagg	264 bp
*sod*	NM_ 017051.2	gacattgtgcctctgggttt	gccctgcatactttgtccat	114 bp
*catalase*	NM_012520.2	gaggaaacgcctgtgtgaga	ttggcagctatgtgagagcc	201 bp
*bax*	NM_0170592	gctggacactggacttcctc	ctcagcccatcttcttccag	168 bp
*bcl-2*	NM_016993.1	tctcatgccaagggggaaac	tatcccactcgtagcccctc	192 bp
*caveolin-3*	NM_019155.2	ggcacggatcatcaaggaca	acacgccatcgaagctgtaa	177 bp
*SERCA2a*	NM_001110139.2	caacacatcttccagccctct	acttggctgatggcttctgtt	246 bp

#### Plasma CK-MB

Plasma samples were thawed and activity levels of CK-MB enzyme were estimated in control and treated samples as per the instruction manual of ENZOPAK CK-MB kit.

#### Microscopic and macroscopic evaluation of cardiac tissue

Tissue samples of ventricle (control and treated) were fixed in 10% buffered paraformaldehyde after autopsy. Later, tissue samples were dehydrated with series of graded alcohol and embedded in paraffin wax. Tissue sections (5 μm) were cut, mounted onto slides and stained with haematoxylin and eosin (HXE) and photographed (Leica DM 2000) at 100X and 400X. Fresh transverse ventricular slices (1–2 mm) were stained with 2,3,5-triphenyltetrazolium chloride (TTC) at 37°C for 20 min and photographed using Canon power S70 shot digital camera [26]. The % infarct area of the ventricles was measured using Image J software (NIH, USA). Whereas, the ventricular thickness was measured using an occulometer [27].

#### Homology modelling and molecular docking

The sequence of Rat ß1 adrenergic receptor (β_1_AR) was retrieved from NCBI sequence database (accession number NP_036833 XP_001063787) and the 3D model was generated using CPHmodels-3.2 Server. Further the stereochemical quality of the modelled structure was evaluated through Ramachandran plot. Molecular docking of delphinidin-3-glucoside and cyanidin-3-glucoside with Rat β_1_AR model was performed using Glide program in Schrodinger and calculations were done using Extra Precision (XP) method. The protein and the ligand molecules were prepared for docking using Protein Preparation Wizard and LigPrep respectively, available in Schrodinger suite. A 20 Å grid box was generated at the active site of the β_1_AR using three active site residues N352, S228 and D138. Information of these three residues was retrieved from the co-crystal structure of quinoline with Turkey β_1_AR (PDB ID: 3ZPR). PyMol was used for visualization of molecular interactions.

#### Statistical analysis

The data were expressed as mean ± SEM and analyzed by one way analysis of variance (ANOVA) using Graph Pad Prism 3.0 (CA, USA). P<0.05 were considered to be significant.

## Results

### Anthocyanin content

**Fig 1:**
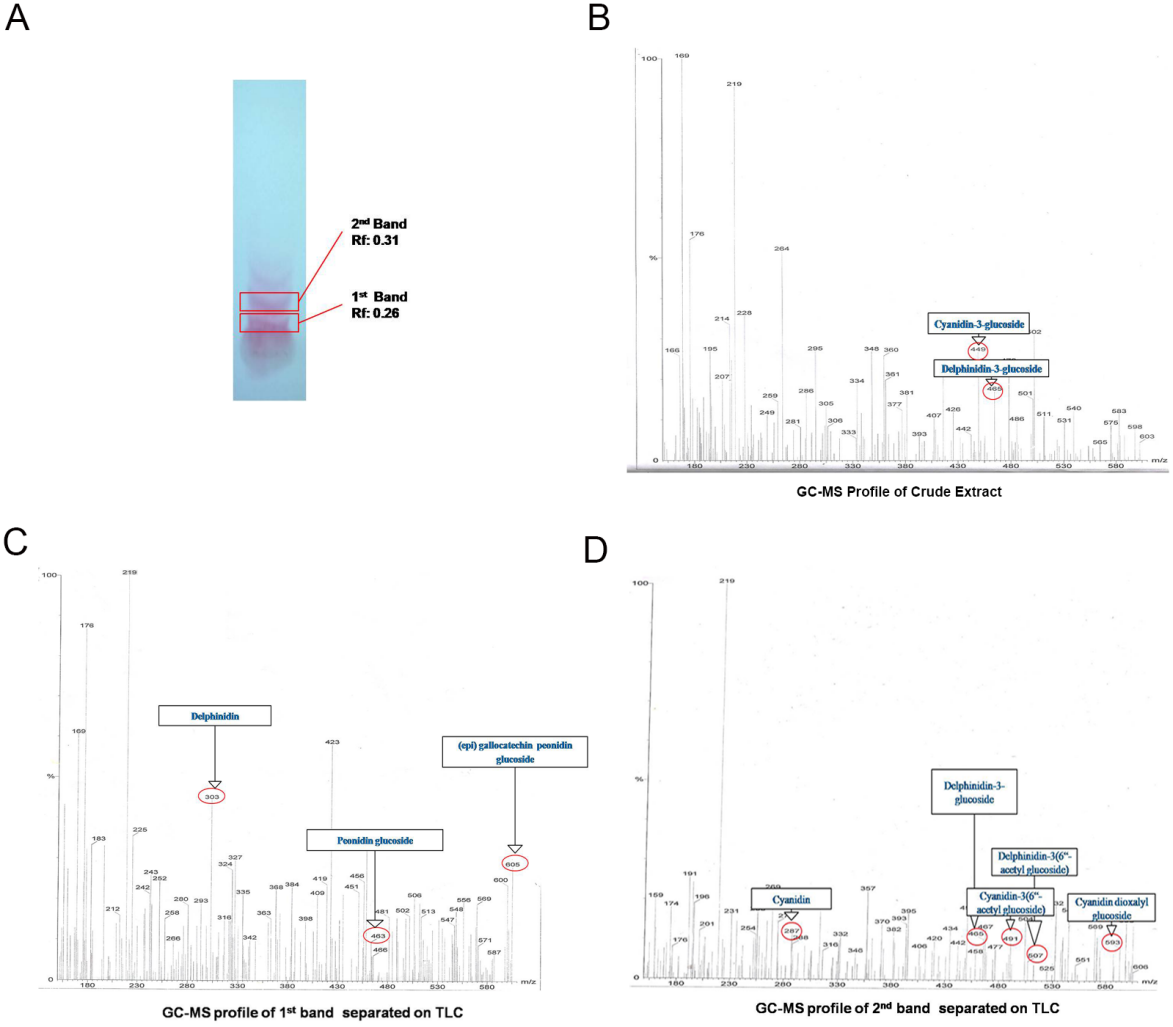
Characterization of ARCE. (A) TLC chromatogram of ARCE separated on aluminium silica gel. The spectra represent GC-MS profiles of (B) crude ARCE, (C) 1^st^ and (D) 2^nd^ bands separated on TLC.

Total anthocyanin content in ARCE was found to be 86.004 ± 3.103 mg/100gm. TLC of ARCE revealed two bands with R_f_ value 0.26 and 0.31 respectively. These values were in the R_f_ value range of 0.2–0.35 and which indicate presence of monoglucosides delphinidin-3-glucoside and cyanidin-3-glucoside [28] (Fig 1A). The GC-MS spectra provided information regarding the structural identification of anthocyanin pigments. The m/z ratio of the daughter and parents ions, confirmed the presence of anthocyanins. Analysis of crude extract (S1A Fig and Fig 1B, Table 2) showed presence of cyanidin-3-glucoside (449 m/z) and Delphinidin-3-glucoside (465 m/z). Whereas, analysis of bands obtained from TLC showed presence of daughter ions of (epi) gallocatechin delphinidin (303 and 481 m/z), (epi) gallocatechin peonidin glucoside (605 m/z), peonidin glucoside (463 m/z) in the first band (S1B Fig and Fig 1C, Table 3) and Cyanidin (287 m/z), Cyanidin-3(6“-acetyl glucoside) (491 m/z), Cyanidindioxalyl Glucoside (593 m/z), Delphinidin-3(6“-acetyl glucoside)(507 m/z) and delphinidin-3-glucoside (465 m/z) in second band (S1C Fig and Fig 1D, Table 4). Overall, presence of cyanidin and delphinidin monoglucosides were recorded in ARCE.

**Table 2:** GC-MS profile of crude extract.

**Peak**	**Retention Time**	**Area**	**Height**	**Area %**
1	9.432	2183807.0	47,715,000	12.32
**Mass Spectrum Profile**				
**Peak**	**Retention Time**	**Daughter fragment**	**Parent fragment**	**Peak identification**
1	9.432	-	449	Cyanidin-3-glucoside
			465	Delphinidin-3-glucoside

**Table 3:** GC-MS profile of 1^st^ band.

**Peak**	**Retention Time**	**Area**	**Height**	**Area %**
1	3.569	706473.9	12,952,837	61.56
**Mass spectrum profile**				
**Peak**	**Retention time**	**Daughter fragment**	**Parent fragment**	**Peak identification**
1	3.569	463	605	(epi) gallocatechin peonidin glucoside
2	3.569	481, 303	-	(epi) gallocatechin delphinidin

**Table 4:** GC-MS profile of 2^nd^ band.

**Peak**	**Retention Time**	**Area**	**Height**	**Area %**
1	3.55	475339.5	8,855,368	41.59
**Mass spectrum profile**				
**Peak**	**Retention Time**	**Daughter fragment**	**Parent fragment**	**Peak identification**
1	3.55	287	491	Cyanidin-3(6“-acetyl glucoside)
			593	Cyanidin dioxalyl glucoside
2	3.55	465	507	Delphinidin-3(6“-acetyl glucoside)

### Cytotoxicity and flowcytometry analysis

**Fig 2:**
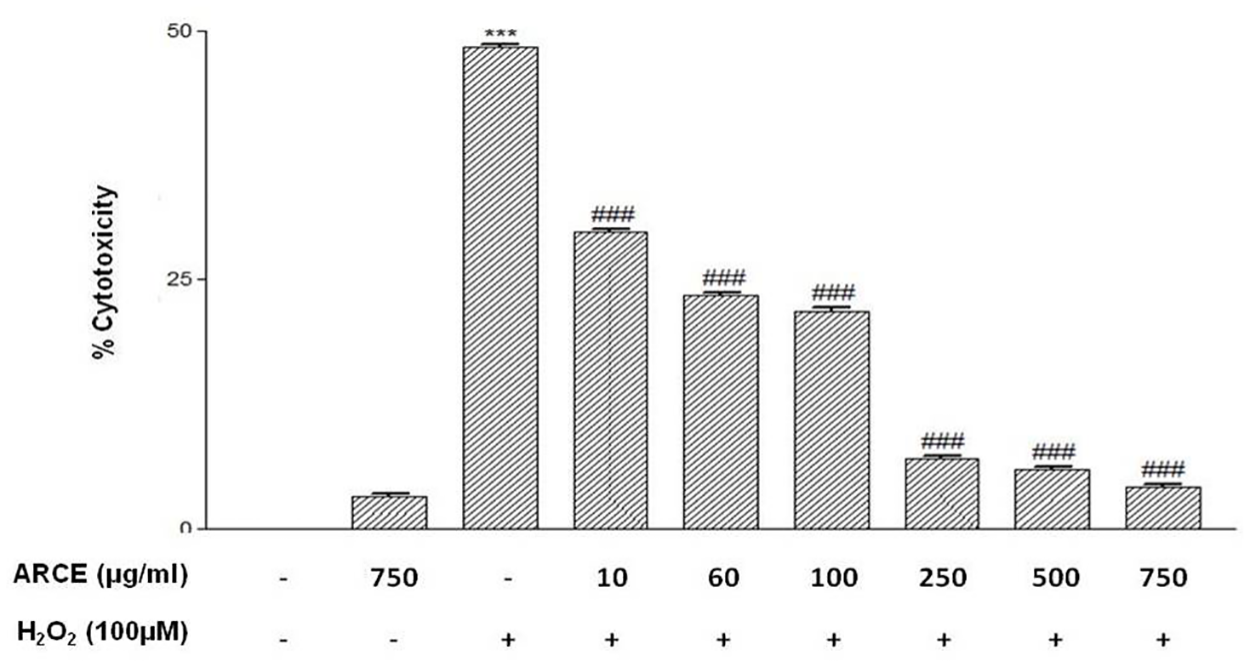
Effect of ARCE on cytotoxicity in H9c2 cells. H9c2 cells were treated with H_2_O_2_ (100 μM) for 12h with or without pretreatment of ARCE (100–750 μg/ml) for 24 h. Untreated cells were used as control. % Cytotoxicity was determined by MTT Assay. The data were represented asmean ± SEM, for three independent experiments. ***P<0.001 vs. control group and ### P<0.001 vs. H_2_O_2_ group.

**Fig 3:**
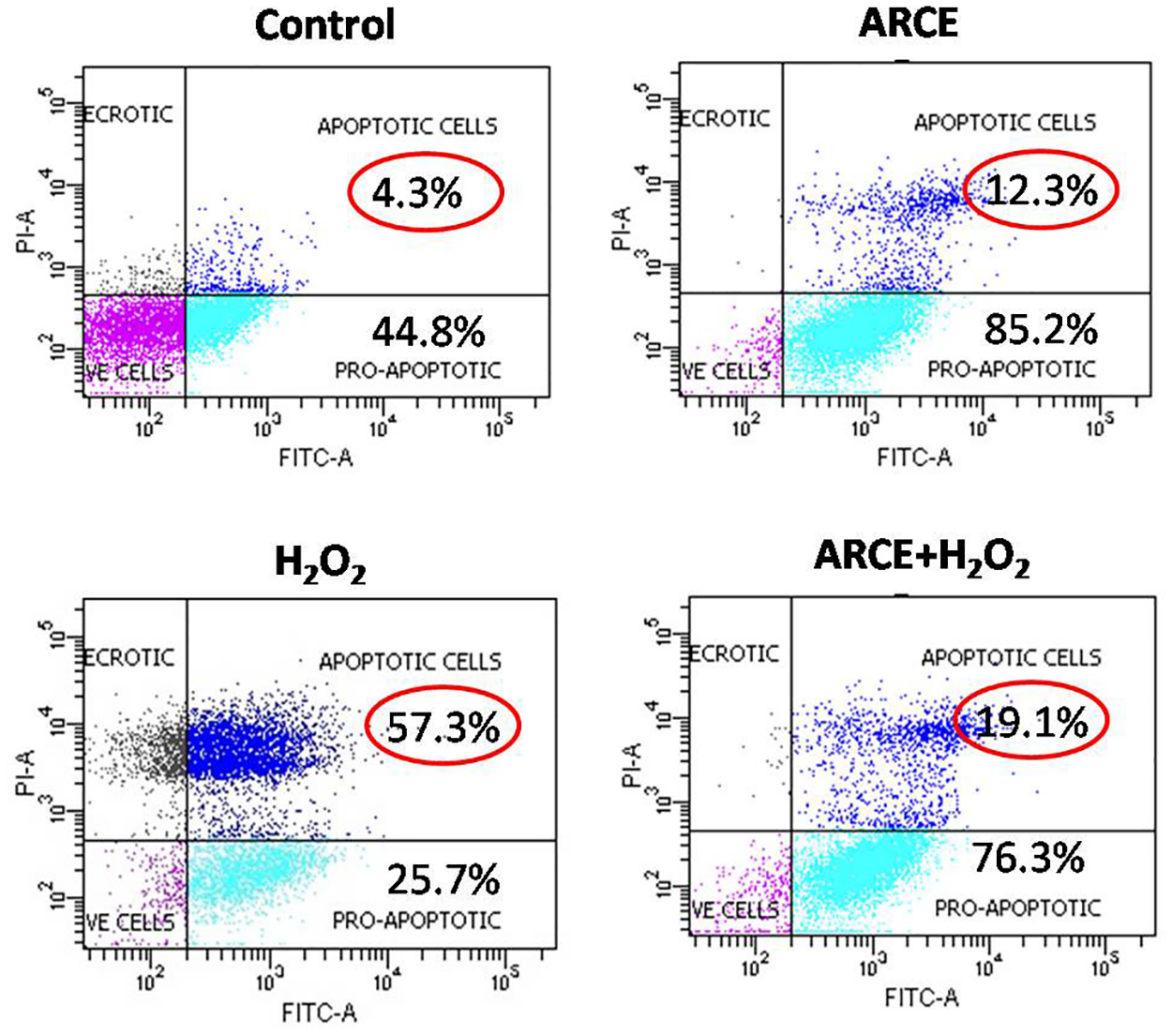
ARCE prevented H_2_O_2_ induced apoptosis in H9c2 cells. The cells were subjected to various treatments followed by staining with Annexin V-Alexa 488/PI for 15 min at 37°C in dark. Untreated cells were used as control. The stained cells were analyzed by flow cytometer. Double positive events indicate apoptotic cells (values encircled in red) and double negative events indicate viable cell population.

ARCE was non toxic but H_2_O_2_ treatment accounted for 48–50% cytotoxicity in H9c2 cells. However, ARCE+H_2_O_2_group accounted for a decrement in cytotoxicity in a dose dependent manner (Fig 2). Also, ARCE treatment accounted for less number of apoptotic cells (12.3%) as compared to H_2_O_2_ treated cells (57.3%). However, ARCE+H_2_O_2_ group accounted for a decrement in apoptosis (19.1%) comparable to that of control or ARCE treated cells (Fig 3)

### Gene expression studies in H9c2 cells

mRNA levels of enzymatic antioxidants (*sod* and *catalase*) were downregulated in H_2_O_2_ treated group, but the same were upregulated in ARCE and ARCE+H_2_O_2_ treated groups and were comparable to that of control group (S2A Fig). mRNA level of proapoptotic gene (*bax*) in H9c2 cells was high in H_2_O_2_ treated group whereas, the same was less in ARCE and ARCE+H_2_O_2_ treated groups. Likewise, anti-apoptotic gene (*bcl-2*) expression was lowered in H_2_O_2_ treated group but was relatively higher in ARCE and ARCE+H_2_O_2_ treated groups (S2B Fig).

### Gene expression studies in rat cardiac tissue

**Fig 4:**
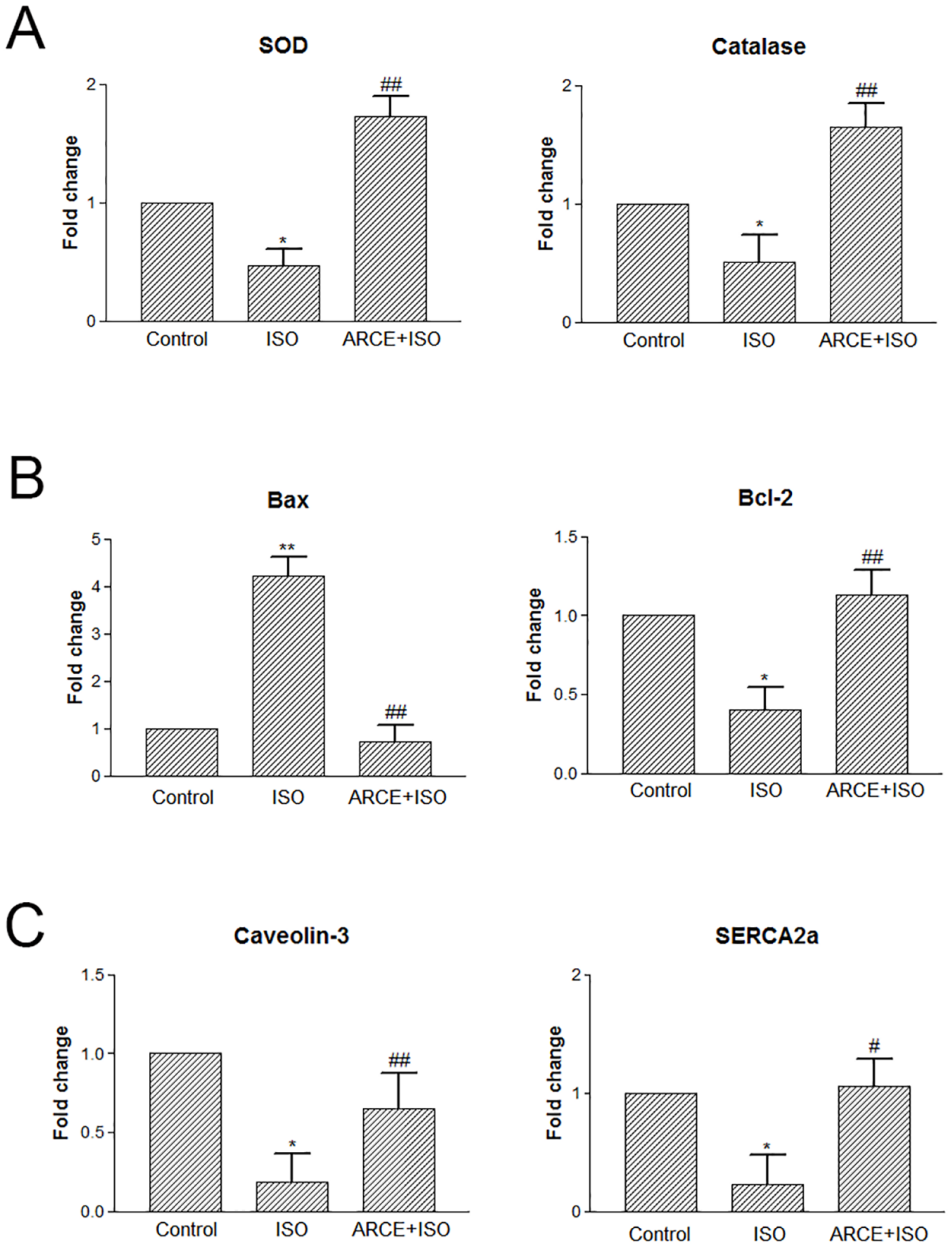
ARCE prevented ISO induced modulations in gene expressions in rat heart tissues. The total RNA isolated from heart tissues from rats of various treatment groups were subjected to cDNA synthesis and followed by quantitative PCR for (A) antioxidant genes (*sod* and *catalase*), (B) pro-apoptotic (*bax*) and anti-apoptotic (*bcl-2*) genes and (C) myocardium specific *caveolin-3* and *SERCA2a* were analysed by quantitative PCR. The data were represented as mean ± SEM, two independent experiments, n = 6 in each experiments. **P<0.01 and *P<0.05 vs. control group; ##P<0.01 and #P<0.05 vs. ISO group.

mRNA levels of intracellular antioxidants (*sod* and *catalase*) were found to be significantly low in ISO treated rats but the same were significantly upregulated in ARCE+ISO group (Fig 4A). Pro-apoptotic gene (*bax*) showed upregulation following ISO treatment whereas, ARCE+ISO treatment could negate the increment with levels comparable to control group. Expression level of anti-apoptotic gene (*bcl-2*) was lower in ISO and higher in ARCE+ISO treated groups, (Fig 4B). mRNA levels of *caveolin-3* and *SERCA2a* show a significant decrement in cardiac tissue following ISO treatment whereas, supplementation of ARCE prevented the said decrement as seen in Fig 4C.

### Microscopic observation and plasma CK-MB

**Fig 5:**
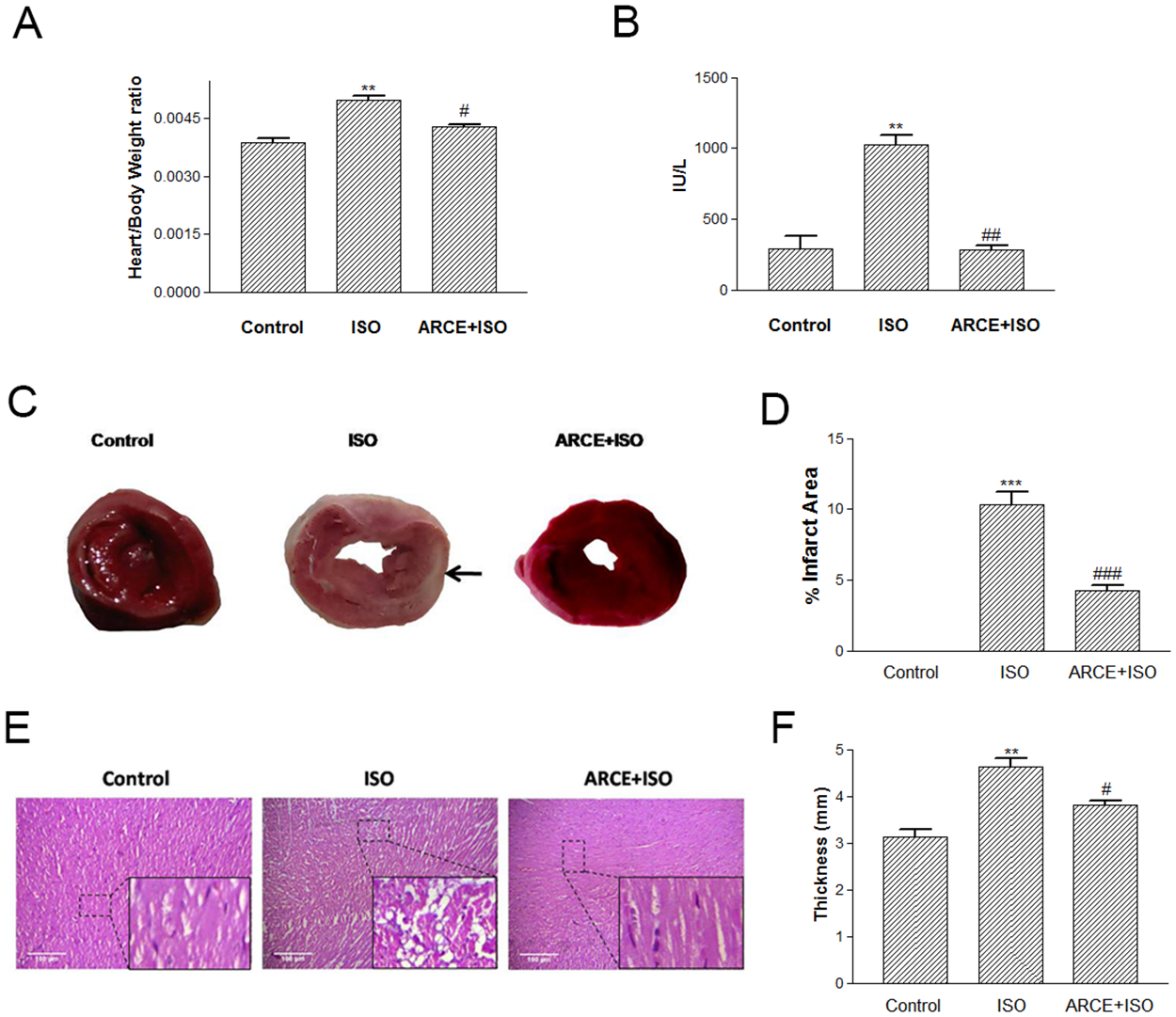
ARCE prevented ISO induced myocardial damage in rats. The plots represents (A) the cardiosomatic indices (heart weight: body weight) and (B) activity levels of CK-MB of control and treated rats. (C) Representative images of the ventricular tissue sections (control and treated) stained with TTC. Arrows indicate infarcted regions. (D) The plot represents % infarct area as measured from the TTC stained sections using Image J software. Further, the ventricular tissue samples were fixed, dehydrated and subjected to paraffin was embedding. Tissue sections were cut and mounted on the slide. (E) Representative images of the ventricular tissue sections stained with HXE. Magnification = 100X (400X for inset). The data were expressed as mean ± SEM from two independent experiments, n = 6 in each experiments. ***P<0.001 and **P<0.01 vs. control group; ###P<0.001, ##P<0.01 and #P<0.05 vs. ISO group.

ISO treatment accounted for significant increment in Heart: Body weight ratio (HW:BW) and plasma CK-MB levels (P<0.01) (Fig 5A and 5B). However, these parameters were comparable to control in ARCE+ISO treated group. TTC stained sections of ventricular tissue of control rats showed brick red color indicating healthy tissue whereas, that of ISO treated rats was pale in colour with white (necrotic) patches. However, necrotic tissue was minimal in ARCE+ISO group (Fig 5C). Image analysis of TTC stained ventricular sections in ISO treated rats showed higher percentage of infarct areas compared to ARCE+ISO treated rats. Control group did not show any infarct tissue (Fig 5D). Ventricles of ISO treated rats showed hypertrophy and accounted for more thickness than the control. However, in ARCE+ISO treated rats, it was comparable to that of control (Fig 5E). Haematoxylin-Eosin stained sections of ventricular tissue of ISO treated rats showed gross derangement of myocardial fibres. Whereas, ARCE+ISO treated group showed intact multinucleated fibres identical to that of control (Fig 5F).

### Homology modelling and molecular docking

**Fig 6:**
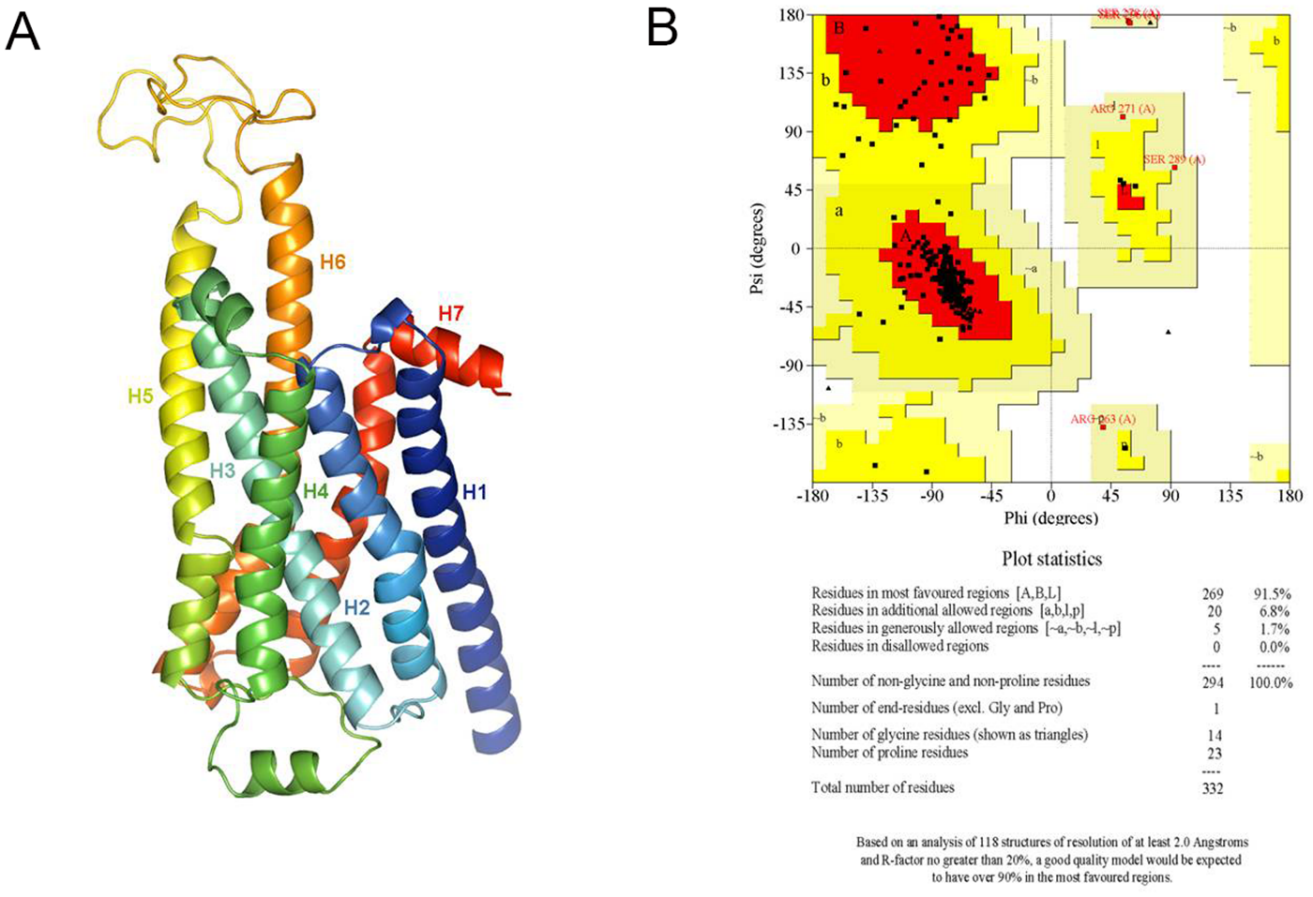
Homology model of β_1_AR. (A) 3D model of Rat β_1_AR showing a bundle structure constitutes of seven helices, the order of helices are marked from N-C terminal as per their respective colours. (B) Ramachandran plot of modelled Rat β_1_ AR showing stereochemical parameters of each residues present in the structure.

**Fig 7:**
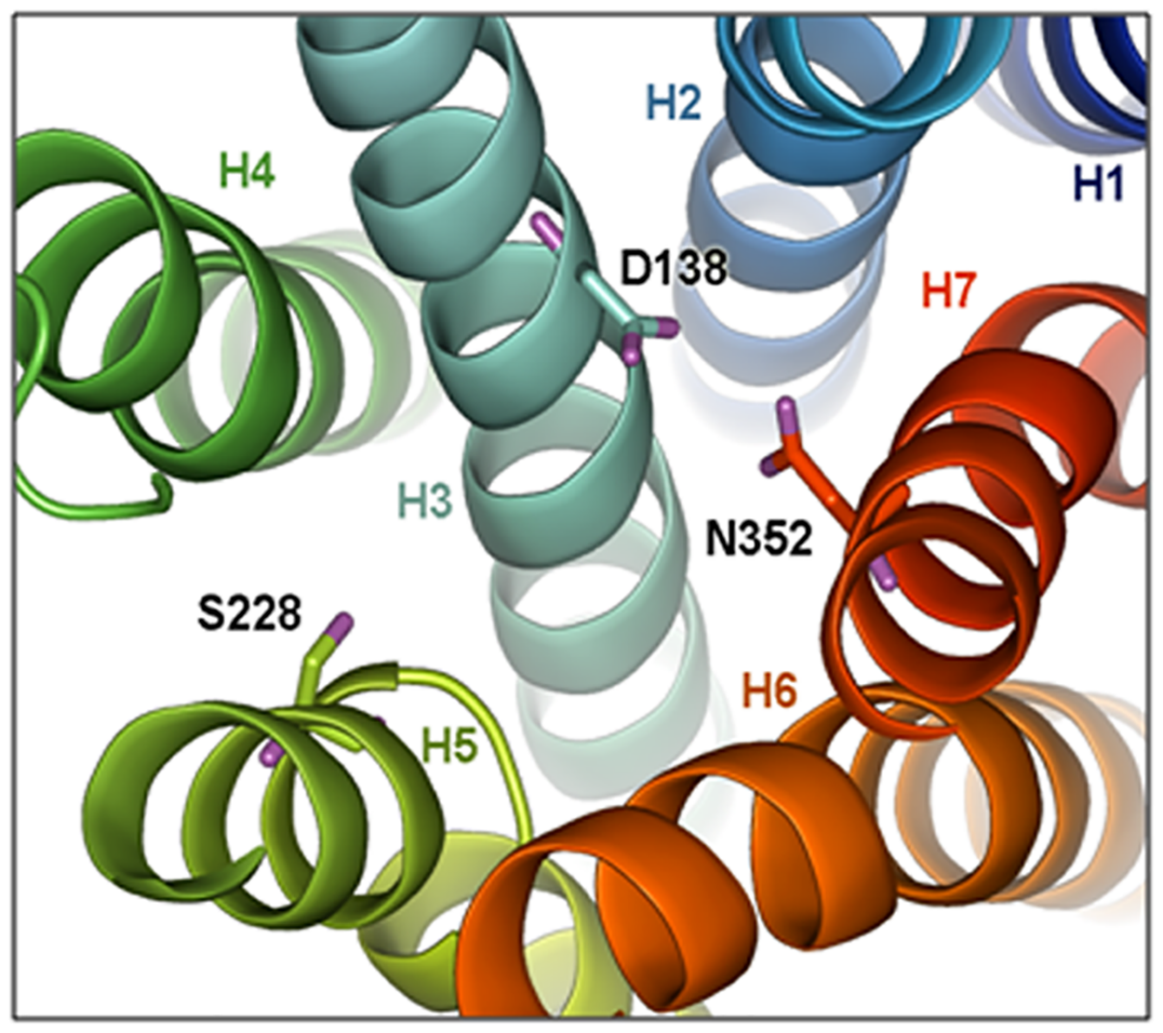
Active site structure of Rat β_1_AR. Three homologous active site residues (D138, S228a and N352) important for binding quinoline as obtained from the co-crystal structure of Turkey β_1_AR (PDB ID: 3ZPR).

**Fig 8:**
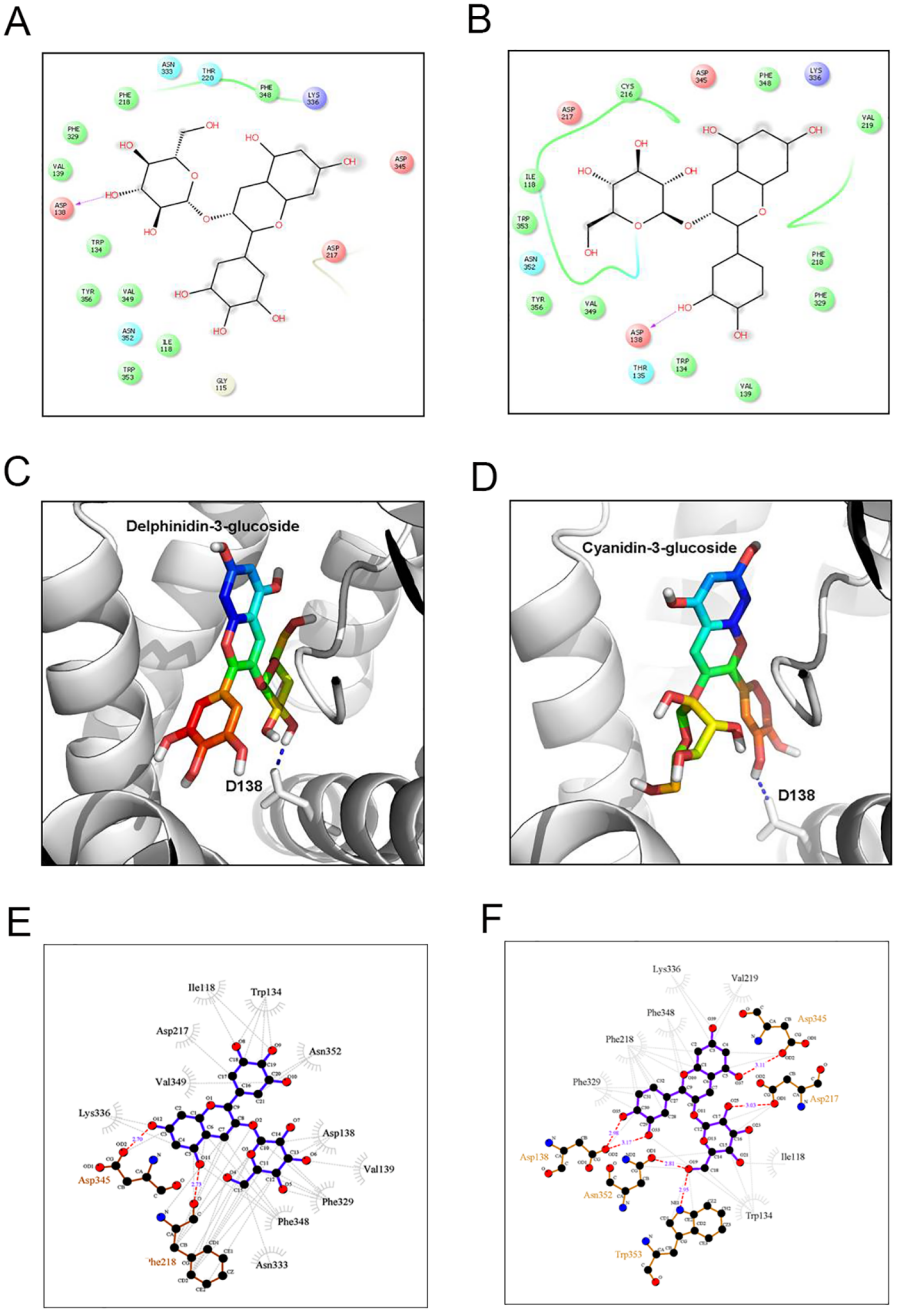
Molecular interaction of delphinidin-3-glucoside and cyanidin-3-glucoside with Rat β_1_ adrenergic receptor. 2D representation of Rat β_1_AR with (A) Delphinidin-3-glucoside and (B) Cyanidin-3-glucoside. Residues in green spheres are hydrophobic, blue spheres are positively charged, cyan spheres are polar, and red spheres are negatively charged. The ligand atoms involved in hydrophobic interactions are marked in gray. The purple arrows and their directions represent hydrogen bonds between the ligand and the protein. 3D representations of the interactions are shown for (C) Delphinidin-3-glucoside and (D) Cyanidin-3-glucoside, the Hydrogen boding with D138 is showing through blue dotted line. (E) LigPlot diagram of Delphinidin-3-glucoside and (F) Cyanidin-3-glucoside.

Thermo-stabilised Turkey β_1_ Adrenergic Receptor bound to quinoline served as a template showing 69.9% alignment with query Rat β_1_AR sequence (S3 Fig). The 3D structure of modelled protein consisted of seven helical structures in bundled formation with flexible loops between Helix-5 (H5) and Helix-6 (H6), residue numbers viz. 258 to 298 (Fig 6A). Ramachandran plot analysis of the model showed that 91.5% residues were in the most favored regions (Fig 6B). Further, D138, S228 and N352 residues in turkey β_1_were found to be conserved in Rat β_1_AR as per the query template alignment (S3 Fig and Fig 7). Molecular docking of Cyanidin-3-glucoside and Delphinidin-3-glucoside with Rat β_1_AR showed that they were well accommodated within the active site and interacted through the hydrophobic and electrostatic bonds at a distance of 2.5 to 3.2 Å. These two anthocyanins accounted for Glide XPG (docking) scores of -8.7 kcal/mol (Fig 8A, 8C and 8E) and -8.5 kcal/mol respectively (Fig 8B, 8D and 8F).

## Discussion

Plant anthocyanins have been extensively studied and reported for their therapeutic properties in human diseases but *in vivo* stability of anthocyanin is always a concern. However, anthocyanins from red cabbage have been reported to have *in vivo* stability as evidenced by the content of its metabolic byproducts detected in urine [29]. Red cabbage is rich in acylated anthocyanins with strong antioxidant activity, stability and therapeutic properties [30]. In our study, red to purple bands obtained in thin layer chromatography of ARCE confirmed presence of anthocyanins [28]. Further, mass spectra obtained by GC-MS revealed presence of cyanidin-3-glucoside and delphinidin-3-glucoside. These results are in agreement with and comparable to the findings of Wiczkowski [30].

Early studies initiated in our lab had reported ARCE mediated decrement in intracellular oxidative stress and restoration of mitochondrial membrane potential in H_2_O_2_ treated H9c2 cells [20]. Also, safety evaluations had revealed that ARCE was non-toxic to H9c2 cells (10–100 μg/ml) and Swiss albino mice (1000–3000 mg/kg) [20, 31]. Keeping these findings as a background, the present study was initiated to decipher the underlying mechanism of ARCE mediated cardioprotection. Intracellular oxidative stress and resultant mitochondrial membrane damage causes release of cytochrome c and activation of intrinsic apoptotic cascade [32, 33]. In the present study, a dose-dependent reduction in H_2_O_2_ induced cytotoxicity and apoptosis of H9c2 cells clearly implicated at ARCE mediated prevention of apoptotic cascade in oxidatively stressed cells. These results are attributable to the free radical scavenging property of ARCE [20] that had accounted for reduced cytotoxicity and prevented trigger of apoptotic cascade.

In rats, isoproterenol had been reported to increase oxygen demand, deplete ATP levels, cause calcium overload and undergo auto-oxidation leading to formation of free radicals [4, 34, 35]. In such a scenario, enzymatic antioxidants (*sod* and *catalase*) have been known to undergo degradation and subsequent exhaustion that furthers the magnimity of oxidative damage within a cell [36]. Therefore, in cardiac tissue ISO induced lipid peroxidation, membrane damage and leakage of CK-MB in plasma are prominent markers of experimentally induced myocardial infarction [37]. In our study, ARCE pretreatment was instrumental in providing cardioprotection as evidenced by Heart: Body weight ratio, decreased circulating levels of CK-MB, improved levels of enzymatic antioxidants (*sod* and *catalase*) and favourable modulations of apoptotic markers (*bax* and *bcl*-2). Cardiac tissue samples of ISO treated rats showed extensive derangement of cardiac syncitium (HXE staining) and prominent infarcted area (TTC staining). These changes were less pronounced in ARCE+ISO group that also corroborates with the higher levels of mRNA of *sod*, *catalase* and *bcl-2* in this group. Hence, ARCE contributes towards imparting overall cytoprotection to the myocardial tissue. Caveolin, a protein that functions as chaperones and forms little caveolae, plays an important role in signal transduction, vesicular transport, regulation of cholesterol and calcium homeostasis [38, 39]. In cardiomyocytes, β_1_ adrenergic receptors are concentrated in the caveolae, wherein ISO has been reported to have more affinity for ß_1_ adrenergic receptor with consequential calcium influx, chronotropic-ionotropic imbalance and hypertrophy [40]. In our study, we had recorded upregulation of *caveolin-3* in ARCE+ISO group. This result is attributable to the membrane stabilizing property of ARCE that has also been reported in a study conducted with erythrocytes [17].

CPH models-3.2 server uses profile-profile alignment method guided by secondary structure and exposure predictions to find out best template structure for model building. Thermostabilised Turkey β_1_ adrenergic receptor bound to quinoline was identified as a template with 69.9% sequence identity with our query Rat β_1_ adrenergic receptor sequence. The active or ligand binding site of Rat β_1_ adrenergic receptor with loop structures and residues (258–298) was unique but, rest of the residues were conserved as per turkey β_1_ adrenergic receptor [41]. Further, molecular docking of cyanidin-3-glucoside and delphinidin-3-glucoside with Rat β_1_ adrenergic receptor showed that they could not only accommodate in the active site, but also could interact through hydrophobic and electrostatic interactions as observed in LigPlot diagram. This further substantiates the effective Glide XPG (docking) scores for cyanidin-3-glucoside and delphinidin-3-glucoside. Hence, these anthocyanins are implied for imparting effective cardioprotection due to the said leads generated from the docking studies.

Previous studies in our lab have reported ISO mediated increase in activity levels of Ca^+2^ ATPase in cardiac tissue of rats [42]. Sarcoplasmic Reticulum Calcium ATPase cardiac isoform 2a (*SERCA2a*) has been reported to play a crucial role in control of spatio-temporal patterns of intracellular calcium signalling. ISO mediated stimulation of B-adrenergic receptor activates protein kinase A (PKA) that phosphorylates calcium channels and increases calcium overload within cytoplasm. Ryanodine receptor (RyR) increases net calcium load in cytoplasm due to its efflux from sarcoplasmic reticulum resulting in muscle contraction. SERCA2a is instrumental in restoring calcium in sarcoplasmic reticulum therefore, decrement in expression of SERCA2a increases cytoplasmic calcium load causing arrhythmia and myocardial damage [43, 44]. This includes altered contraction, hypertrophic growth and apoptosis of cardiomyocytes that have been known to be reverted by upregulation of *SERCA2a*. Hence, merits of *SERCA2a* are also debated as a pharmacotherapeutic target in preventing myocardial infarction [45]. In the present study, ISO treated rat recorded decrement in *SERCA2a*expression whereas; the ones pre-treated with ARCE showed restored *SERCA2a* levels. These observations are significant and add a new dimension to ARCE-*SERCA2a* crosstalk in infarcted myocardium.

## Conclusion

It can be concluded that ARCE manifests multipronged therapeutic effects viz. improving the status of intracellular antioxidants, preventing membrane damage and apoptosis. Also, experimental evidences on alleviation of ISO induced modulations of *caveolin-3* and *SERCA2a* in cardiac tissue by ARCE explains its cardioprotective potential. Molecular docking scores of cyanidin-3-glucoside and delphinidin-3-glucoside provide insights on their stable interaction with β_1_ adrenergic receptor and ARCE mediated prevention of myocardial damage. However, lack of information on the crystal structure of rat β_1_ adrenergic receptor is a possible limitation of the present study. Also, efficacy of ARCE as a cardioprotectant assessed herein is restricted to male rats. A similar experiment with estrogen deficient female rats or variation in age may result in a differential response that needs to be ascertained. Nevertheless, this report throws light on the underlying mechanism of ARCE induced cardioprotection.

## Supporting information

S1 Fig GC chromatograms of anthocyanin pigments from (A) crude extract, (B) 1^st^ and (C) 2^nd^ bands separated on TLC. The peaks marked in green represent those subjected to further characterization by MS.
(TIF)

S2 Fig ARCE prevented H_2_O_2_ mediated antioxidant depletion and apoptotic changes in H9c2 cells. Total RNA was isolated using TRIzol reagent from treated and control cells and the mRNA levels of (A) antioxidant genes (*sod* and *catalase*) and (B) pro-apoptotic (*bax*) and anti-apoptotic (*bcl-2*) genes were analysed by quantitative PCR. The data were represented as mean ± SEM, for three independent experiments. **P<0.01 and *P<0.05 vs. control group; #P<0.05 vs. H_2_O_2_ group.
(TIF)

S3 Fig Alignment of Rat β_1_AR (residue 49 to 380) with the template sequence of Turkey β_1_AR (PDB ID: 3ZPR) showing critical residues for quinoline binding marked within box. The actual positions of these residues in Turkey β_1_AR are labelled.
(TIF)
