# Supplementary figures and images for: Anthocyanin rich extract of Brassica oleracea L. alleviates experimentally induced myocardial infarction
Source: PLoS One. 2017 Aug 1;12(8):e0182137. doi: 10.1371/journal.pone.0182137 (PMC5538674; doi:10.1371/journal.pone.0182137)

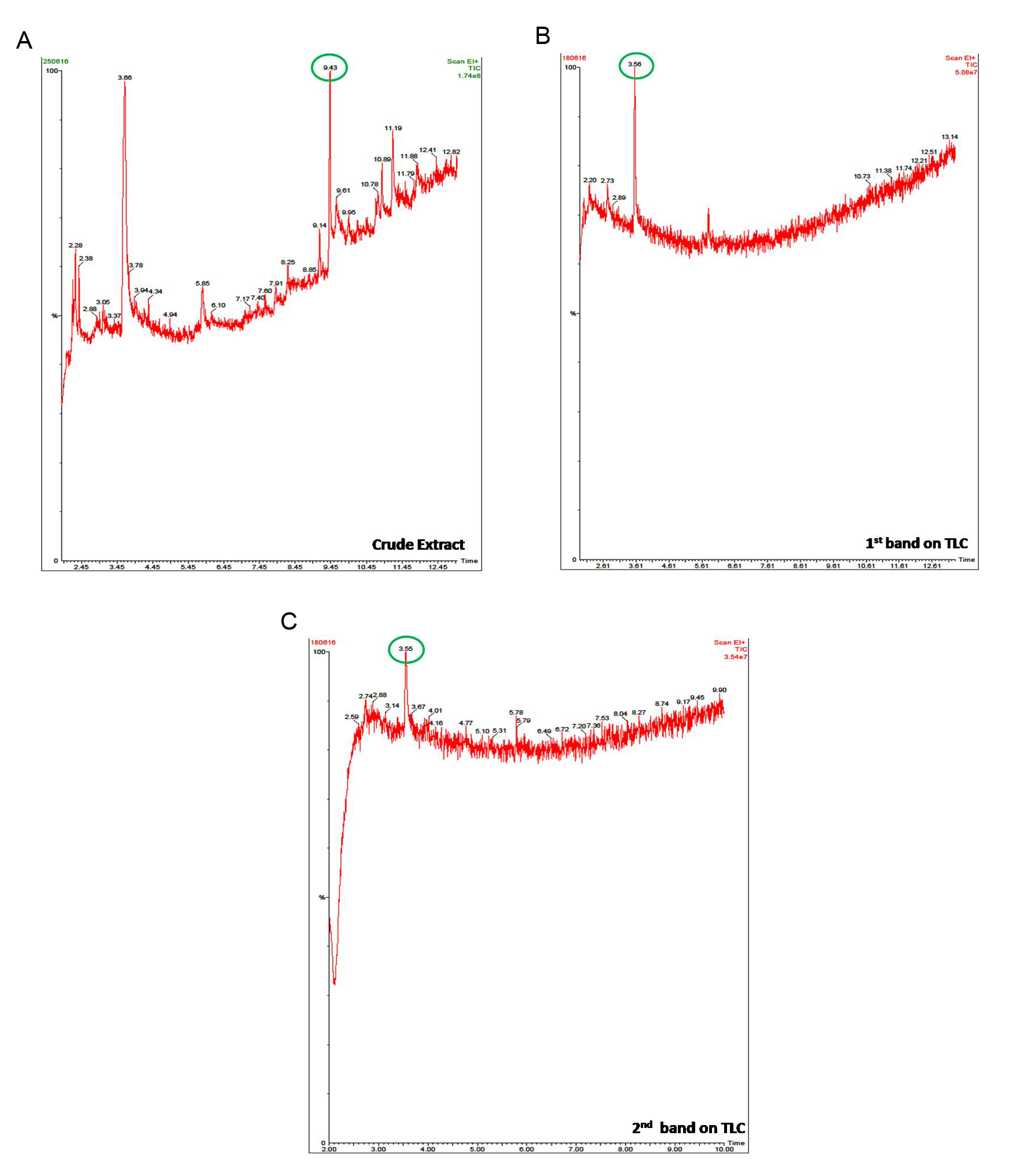

Supplement: S1 Fig — The peaks marked in green represent those subjected to further characterization by MS. (TIF) [file pone.0182137.s001.tif]

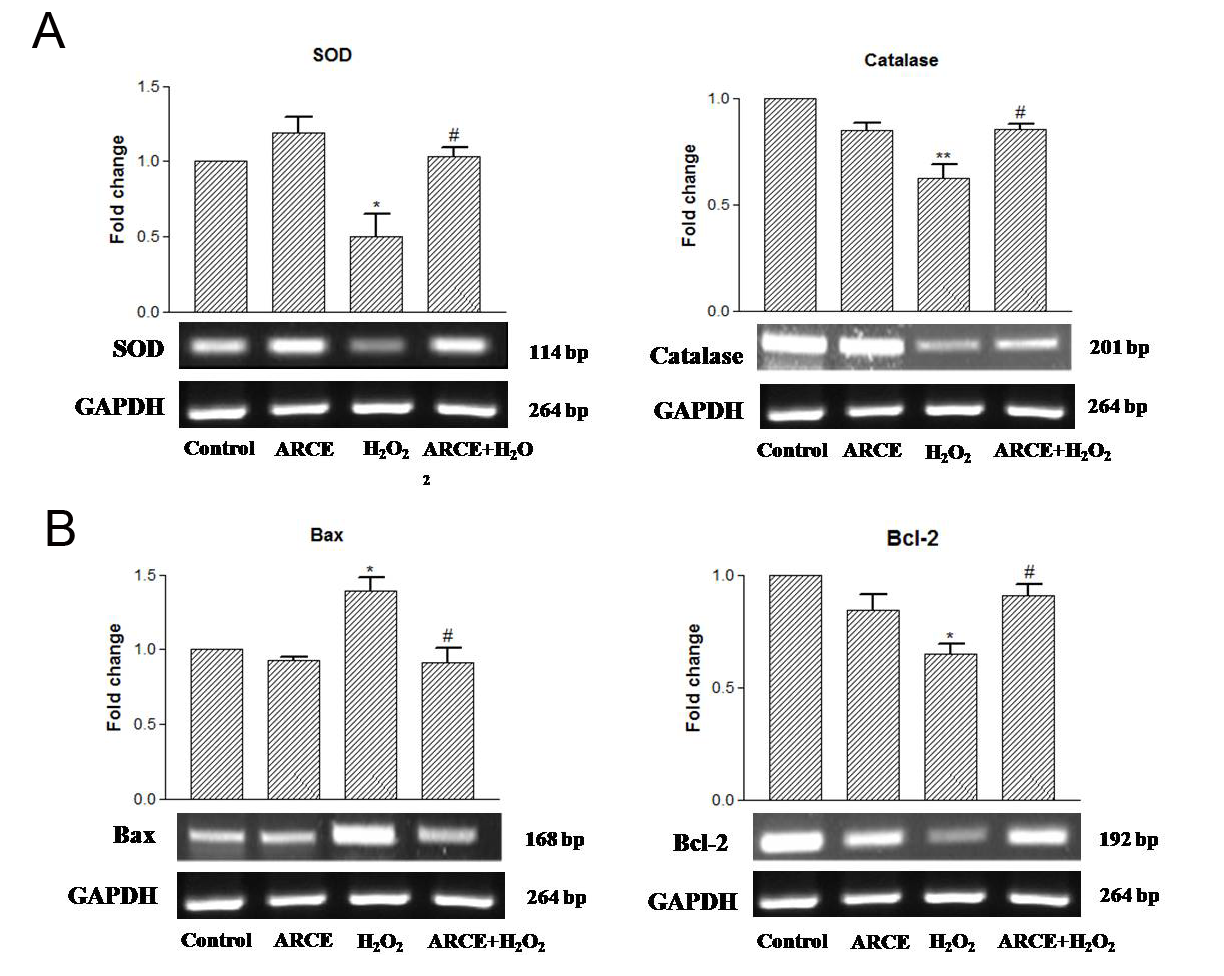

Supplement: S2 Fig — Total RNA was isolated using TRIzol reagent from treated and control cells and the mRNA levels of (A) antioxidant genes (sod and catalase) and (B) pro-apoptotic (bax) and anti-apoptotic (bcl-2) genes were analysed by quantitative PCR. The data were represented as mean ± SEM, for three independent experiments. **P<0.01 and *P<0.05 vs. control group; #P<0.05 vs. H2O2 group. (TIF) [file pone.0182137.s002.tif]

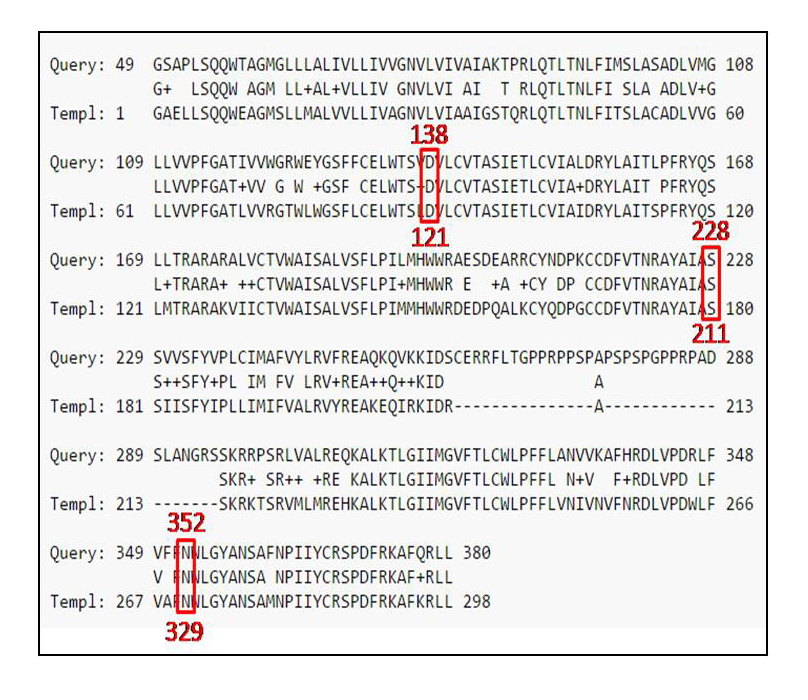

Supplement: S3 Fig — The actual positions of these residues in Turkey β1AR are labelled. (TIF) [file pone.0182137.s003.tif]
